# Study on Spatial Spillover Effects of Logistics Industry Development for Economic Growth in the Yangtze River Delta City Cluster Based on Spatial Durbin Model

**DOI:** 10.3390/ijerph14121508

**Published:** 2017-12-04

**Authors:** Xinxing Xu, Yuhong Wang

**Affiliations:** School of Business, Jiangnan University, Wuxi 214122, China; zmsn_1993@163.com

**Keywords:** logistics industry, economic growth, spillover effect, overall entropy method, spatial durbin model

## Abstract

The overall entropy method is used to evaluate the development level of the logistics industry in the city based on a mechanism analysis of the spillover effect of the development of the logistics industry on economic growth, according to the panel data of 26 cities in the Yangtze River delta. On this basis, the paper uses the spatial durbin model to study the direct impact of the development of the logistics industry on economic growth and the spatial spillover effect. The results show that the direct impact coefficient of the development of the logistics industry in the Yangtze River Delta urban agglomeration on local economic growth is 0.092, and the significant spatial spillover effect on the economic growth in the surrounding area is 0.197. Compared with the labor force input, capital investment and the degree of opening to the world, and government functions, the logistics industry’s direct impact coefficient is the largest, other than capital investment; the coefficient of the spillover effect is higher than other control variables, making it a “strong engine” of the Yangtze River Delta urban agglomeration economic growth.

## 1. Introduction

The logistics industry is the “big artery” of the national economy, and plays an important role in the growth of the national economy. Regional logistics, as an important carrier of regional economy in terms of breaking down the barriers between regions, communication production and consumption, and promoting the coordinated development of regional economy, have an important impact. In recent years, the relationship between the logistics industry and economic growth has attracted the attention of many scholars. The present research was focused on the study of the one-way and two-way relationship between the logistics industry and economic growth.

In the study of bi-directional relations, Li et al. (2010) studied the correlation between logistics capacity and economic growth by using the typical correlation analysis method based on the panel data of 31 provinces and cities in China, and obtained a high correlation between the two [1]. Liu (2011) established a dynamic coupling model from the perspective of system dynamics to analyze the dynamic relationship of the regional logistics industry and economic growth, concluding that coordination could not be achieved in the short term, but that the situation would evolve towards harmony in the long term [2]. Zhang et al. (2014) measured the spatio-temporal coupling of logistics and the economy in Anhui cities through the geographical contact rate and the center of gravity model. The research indicates that there is an obvious coupling relationship between time and space [3]. Zhang et al. (2015) explored the relationship between the logistics industry and the coordinated development of economy, with 21 cities in China as the object of study, by using the gray correlation method, concluding that, compared to other cities, the eastern cities had the highest degree of synergy [4]. Lan et al. (2016) found that the logistics industry infrastructure investment would have a significant impact on the relationship of the coordinated development between the logistics industry and urban economy by using Bayesian network [5]. Wang et al. (2017) used the Tapio model to analyze the decoupling relationship between carbon emissions of transportation and economic growth in Jiangsu Province, found that the decoupled state had a distinct periodic characteristic [6].

In the study of unidirectional relations, Meersman et al. (2017) argued that the investment in transport infrastructure contributes to the growth of the Belgian economy based on the overall growth model and causal factor analysis [7]. Kim et al. (2015) argued that port logistics would have a significant positive impact on the port city economy [8]. Mohmand et al. (2016), based on empirical data from Pakistan, used cointegration analysis and Granger causality tests to arrive at a single causal relationship between investment in transport infrastructure and economic growth over a long period of time [9]. Tsekeris (2016) used the panel model to measure the impact of domestic transportation on export trade in Greece, concluding that the spillover effect of transportation on the regional export trade is obvious [10]. Kumar et al. (2017) argued that most of the logistics industry agglomeration concentrated in the metropolitan area, and the agglomeration of transport structure will have a significant impact on employment and economy [11]. Juozapaitis et al. (2017) analyzed the feasibility of promoting logistics agglomeration in Lithuania, and explained the important influence of logistics agglomeration on regional economic growth [12]. Cao et al. (2015) obtained that regional logistics had a significant role in promoting economic growth by using China’s provincial panel data to analyze the relationship between the logistics industry and economic growth [13].

Through the combing of the above literature, there are few studies on the spillover effect of the regional logistics industry on the economic growth from the space perspective, whether in one-way relationship or two-way relationship studies. Therefore, this paper first analyzes the mechanism of the logistics industry’s spillover effect on economic growth, followed by the overall entropy method to evaluate the level of regional logistics development, and finally, the spatial durbin model is used to measure the effect of space spillover on the economic development of the Yangtze River Delta urban agglomeration.

## 2. Spillover Effect Model Construction of the Logistics Industry in Relation to Economic Growth

### 2.1. Analysis of the Spillover Mechanism in the Development of the Logistics Industry in Relation to Economic Growth

Transportation, as an important part of logistics activities, not only contributes to the transport revenue of local logistics enterprises, but also to the rational flow of factors between the regions. The development of a region’s traffic means that the central city can deliver the production factors to the surrounding areas, which is conducive to reducing the cost of production in the surrounding areas, and promoting regional economic growth. At the same time, in order to reduce logistics costs, some areas begin to gradually abandon the ideas of their own development in order to achieve co-operation and the introduction of a series of related policies, which attracts cross-regional investment and the construction of logistics infrastructure, regional logistics information, platform docking, etc. To a certain extent, this strengthens the regional exchange of information and economic exchange, and expands the market space, promotes regional economic growth, and has a spillover effect on the economic growth of surrounding areas. 

### 2.2. The Modeling Process of the Spillover Effect on Logistics Industry in Relation to Economic Growth

#### 2.2.1. Evaluation on the Development Level of the Logistics Industry

The measure of direct impact and spatial spillover effect of the development of the logistics industry on economic growth relies on the reasonable evaluation of the development level of the regional logistics industry. On the basis of studies of Tang et al. (2015) [14] and Cai et al. (2016) [15], the rationality and availability of index data and the characteristics of the target regions are considered in this paper; a comprehensive evaluation index system of logistics industry development level in Yangtze River delta is constructed from four aspects: industrial scale, infrastructure, human resources, and industry support. The specific index system is shown in Table 1.

Given that the entropy method is an objective evaluation method based on the index variability, it has been widely used in the evaluation of the development level of the logistics industry [16,17]. However, the traditional entropy method has a limitation. It can only be used to analyze two-dimensional data tables including regions and time points, and can not analyze three-dimensional data tables including indices, time points, and regions. Therefore, this paper draws on the overall entropy methods used by Pan et al. (2015) [18] to evaluate the development level of logistics industry in urban agglomeration on the basis of above index system. Specific steps are shown below in Steps 1–6.

Step 1: According to the n evaluation indices of m regions and t years, the measurement of the development level of the logistics industry is carried out, and the t-section data tables are arranged in chronological order to construct a judgment matrix *p* of mt×n, which is shown as Formula (1),

(1) $$p={\left(x_{ij}^{t}\right)}_{mt\times n}$$

Step 2: The judgment matrix p is normalized.

(2) $${\left(x_{ij}^{t}\right)}^{\prime }=\;\frac{x_{ij}^{t}-x_{j\min}}{x_{j\max}-x_{j\min}}\times 99+1\;,\;1\leq i\leq m,1\leq j\leq n,1\leq t\leq T$$

(3) $${\left(x_{ij}^{t}\right)}^{\prime }=\;\frac{x_{j\max}-x_{ij}^{t}}{x_{j\max}-x_{j\min}}\times 99+1\;,\;1\leq i\leq m,1\leq j\leq n,1\leq t\leq T$$

In Formulas (2) and (3), ${\left(x_{ij}^{t}\right)}^{\prime }\in \left[1,100\right]$ represents the normalized value, $x_{j\min}$ and $x_{j\max}$ represent, the minimum and maximum, respectively of the $j^{th}$ index. The positive indicator should be standardized by Formula (2); otherwise, it is standardized by Formula (3).

Step 3: Calculate the $j^{th}$ indicator of entropy.

(4) $$e_{j}=-\frac{1}{\ln mT}\sum_{t=1}^{T}\sum_{i=1}^{m}\left[\frac{{\left(x_{ij}^{t}\right)}^{\prime }}{\sum_{t=1}^{T}\sum_{i=1}^{m}{\left(x_{ij}^{t}\right)}^{\prime }}\ln\left(\frac{{\left(x_{ij}^{t}\right)}^{\prime }}{\sum_{t=1}^{T}\sum_{i=1}^{m}{\left(x_{ij}^{t}\right)}^{\prime }}\right)\right]$$

Step 4: Calculate the difference coefficient of the $j^{th}$ indicator.

(5) $$g_{j}=1-e_{j}$$

Step 5: Calculate the weight of the $j^{th}$ indicator.

(6) $$w_{j}=\frac{g_{j}}{\sum_{j=1}^{n}g_{j}}$$

Step 6: Calculate the level of development of logistics industry.

(7) $$F_{i}^{t}={\sum_{j=1}^{n}w_{j}\left(x_{ij}^{t}\right)}^{\prime }$$

#### 2.2.2. Spatial Autocorrelation Test

On the basis of the above steps, the spatial autocorrelation of the logistics industry and the spatial autocorrelation of regional economic growth are tested by using the spatial statistical data box in ArcGIS10.2 software (Redlands, CA, USA); the test of Moran’s Index is used to determine necessity of introducing the spatial econometric model. When the Moran’s Index of the explanatory variable exists and can be tested by the significance test, it is necessary to introduce the spatial econometric model, because the variable does not satisfy the classical hypothesis of a homogeneous distribution. The Moran’s Index is shown in Formula (8),

(8) $$Moran\prime s\;I=\frac{n\sum_{i=1}^{n}\sum_{j=1}^{n}w_{ij}\left(x_{i}-\overset{-}{x}\right)\left(x_{j}-\overset{-}{x}\right)}{\left(\sum_{i=1}^{n}\sum_{j=1}^{n}w_{ij}\right)\sum_{i=1}^{n}{\left(x_{i}-\overset{-}{x}\right)}^{2}}$$

where $x_{i}$ and $x_{j}$ represent the observed values of region i and region j, respectively, n is the number of objects to be observed, $\overset{-}{x}=\frac{1}{n}\sum_{i=1}^{n}x_{i}$, and w is the spatial weight matrix. This paper uses the row-standardized classical 0–1 matrix to conduct the study. The matrix form is shown in Formula (9),

(9) $$W=\left[\begin{array}{cccc} W_{11} & W_{12} & \cdots & W_{1n}\\ W_{21} & W_{22} & \cdots & W_{2n}\\ \vdots & \vdots & \cdots & \vdots \\ W_{n1} & W_{n2} & \cdots & W_{nn} \end{array}\right]$$

Except that the diagonal elements of the matrix are all 0; when the region i is adjacent to the region j, $W_{ij}=1$ and when the region i is not adjacent to the region j, $W_{ij}=0$. The range of Moran’s Index converges approximately to [−1,1], as the 0–1 spatial weight matrix is row-standardized [19]. When the Moran’s Index is positive, it indicates that there is a spatial positive correlation, and there is a spatial negative correlation when the value is negative, while the greater the absolute value of Moran’s Index, the greater the degree of spatial correlation. Confirm that “index“ was intended here.

#### 2.2.3. Spatial Spillover Effect Measuring Model Construction and Relevant Testing Steps

Taking into account the impact of the regional logistics industry on the economic growth of local and surrounding areas, the spatial durbin model (SDM) fits both theory and reality more well. Of course, based on rigorous consideration, this paper will also construct a spatial lag model (SAR) and spatial error model (SEM), and illustrate the selection of spatial econometric models and relevant testes. At the same time, in order to overcome the possible endogenous problems of the model, this paper takes the time lag of all the explanatory variables as its surrogate variables, and takes the natural logarithm of variables to reduce possible heteroscedasticity problems. Thus, the spatial econometric models based on the C–D production function are shown in Equations (10)–(12),

(1) Spatial Lag Model (SAR) is mainly used to study the spatial spillover effect of dependent variables on the surrounding areas.
  (10) $$\begin{array}{ll} \ln GDP_{it}= & \rho W\ln GDP_{it}+\beta_{1}\ln WL_{it-1}+\beta_{2}\ln L_{it-1}+\beta_{3}\ln Inv_{it-1}+\beta_{4}\ln Open_{it-1}\\ & +\beta_{5}\ln Gov_{it-1}+\eta_{i}+\delta_{t}+\varepsilon_{it} \end{array}$$
(2) Spatial Error Model (SEM) mainly concentrates on the spatial interaction effect of the missing items in the modeling process.
  (11) $$\begin{array}{l} \begin{array}{ll} \ln GDP_{it}= & \beta_{1}\ln WL_{it-1}+\beta_{2}\ln L_{it-1}+\beta_{3}\ln Inv_{it-1}+\beta_{4}\ln Open_{it-1}\;+\beta_{5}\ln Gov_{it-1}\\ & +\eta_{i}+\delta_{t}+\varepsilon_{it} \end{array}\\ \varepsilon_{it}=\lambda W\varepsilon_{it}+\varphi_{it}\;\varphi_{it}∼N\left(0,\sigma_{it}^{2}I_{n}\right) \end{array}$$
(3) Spatial Error Model (SDM) takes both the spatial lag items of explanatory and explanatory variables into account.
  (12) $$\begin{array}{ll} \ln GDP_{it}= & \rho W\ln GDP_{it}+\beta_{1}\ln WL_{it-1}+\beta_{2}\ln L_{it-1}+\beta_{3}\ln Inv_{it-1}+\beta_{4}\ln Open_{it-1}\;+\\ & \beta_{5}\ln Gov_{it-1}\;+\theta_{1}W\ln WL_{it-1}\;+\theta_{2}W\ln L_{it-1}+\theta_{3}W\ln Inv_{it-1}+\theta_{4}W\ln Open_{it-1}\;\\ & +\theta_{5}W\ln Gov_{it}{}_{-1}+\eta_{i}+\delta_{t}+\varepsilon_{it} \end{array}$$

where GDP stands for regional economic growth, WL represents the level of development of the logistics industry, L represents labor input, Inv represents material capital investment, Open represents the level of opening to the outside world, Gov represents government function, W is 0–1 spatial weight matrix, ρ is spatial autoregressive coefficient reflects the effect of the spatial hysteresis on the explained variables. λ is the spatial autocorrelation coefficient of the error term. β and θ are the regression coefficients and the spatial correlation coefficients, respectively, of the explanatory variables, $\eta_{i}$ is the fixed-space effect, $\delta_{t}$ is the fixed-time effect, $\varepsilon_{it}$ is the random error term.

After building the spatial econometric model, a series of related model tests are necessary to ensure the reliability of the regression results. The main test steps are shown as Steps 1–3.

Step 1: Before the regression analysis, in order to avoid serious multiple collinearity problems between the variables, the correlation coefficient matrix is provided and Variance Inflation Factor (VIF) of each variable is calculated. If the VIF is less than the empirical value of 10, the model can basically be judged to be free of serious multicollinearity problems.

Step 2: According to the non-space panel model, the likelihood ratio test (LR) is used to investigate the existence and significance of the time and space fixed in the model. At the same time, according to the Lagrange Multiplier test (LM) and Robust Lagrange Multiplier test (Robust LM), it is further judged whether there exist spatial lag or spatial error terms in the model. If the null hypothesis that there is no space lag or space error is rejected, LM-lag is superior to LM-error, while Robust LM-lag test is superior to Robust LM-error; in this case, the SAR model should be selected instead of SEM model [20].

Step 3: If the above test indicates the necessity of the inclusion of the spatial factor, the applicability of the SDM model will be further evaluated using the Wald and LR tests. If the null hypotheses $H_{0}:\theta =0$ and $H_{0}:\theta +\rho \beta =0$ are rejected, it is shown that the SDM model can not be simplified to the SAR or the SEM. Therefore, the SDM model is better able to describe the relationship between the development of the logistics industry and economic growth in the Yangtze River Delta urban agglomeration.

On the basis of the above tests, the spatial econometric models will be estimated by the maximum likelihood estimation (ML) [21], the spatial spillover effect of the explanatory variables is measured by the partial differential method proposed by Lesage et al. [22]. The model is introduced into the inverse matrix to transform the SDM model as shown in Formula (13),

(13) $$Y={\left(1-\rho W\right)}^{-1}+{\left(1-\rho W\right)}^{-1}\left(X\beta +WX\theta \right)+{\left(1-\rho W\right)}^{-1}\varepsilon$$

The average effect of the kth explanatory variable is obtained by *D*th extraction, forming a partial differential matrix equation, as shown in Formula (14),

(14) $${\left[\frac{\partial Y}{\partial X_{1k}}\;\cdots \;\frac{\partial Y}{\partial X_{nk}}\right]}_{t}=\left[\begin{array}{ccc} \frac{\partial Y}{\partial X_{1k}} & \cdots & \frac{\partial Y_{n}}{\partial X_{nk}}\\ \cdots & \cdots & \cdots \\ \frac{\partial Y_{n}}{\partial X_{1k}} & \cdots & \frac{\partial Y_{n}}{\partial X_{nk}} \end{array}\right]={\left(I-\rho W\right)}^{-1}\left[\begin{array}{cccc} \beta_{k} & W_{12}\theta_{k} & \cdots & W_{1n}\theta_{k}\\ W_{21}\theta_{k} & \beta_{k} & \cdots & W_{2n}\theta_{k}\\ \cdots & \cdots & \cdots & \cdots \\ W_{n1}\theta_{k} & W_{n2}\theta_{k} & \cdots & \beta_{k} \end{array}\right]$$

Among them, the mean value of the diagonal elements of the matrix represents the direct effect of the explanatory variables, and the mean of the diagonal elements represents the spatial spillover effect of the explanatory variables.

## 3. An Empirical Study on the Spatial Spillover Effect of Logistics Industry Development on Economic Growth

### 3.1. Variable Selection and Data Description

The overall entropy method is used to calculate the data, with logistics industry development level (WL) as the core explanatory variable of the model. Regional economic growth, as the dependent variable, is shown by the use of regional gross domestic product (GDP), and the year 2005 as the base period using the GDP index to remove the impact of price factors. Labor input (L) is indicated by the number of employees employed in the three industries. Material Capital Investment (Inv) is expressed by the proportion of fixed-asset investment to GDP. The level of the opening-up (Open) is expressed by the proportion of total imports and exports to GDP, and the import and export data of *the China City Statistical Yearbook* are converted into RMB according to the annual average exchange rate in US dollars. The role of government functions (Gov) is expressed by the proportion of science & technology and education expenditure to public expenditure.

The statistical data comes from the 2005–2015 *China City Statistical Yearbook* and the Statistical Yearbook of Cities, and the average annual exchange rate is from the State Administration of Foreign Exchange official website.

### 3.2. Evaluation of the Development Level of the Logistics Industry

Based on the panel data of 26 cities in the Yangtze River Delta from 2005 to 2015, the paper uses the steps of the overall entropy method to evaluate the development level of the logistics industry in the urban agglomeration and obtain the level of development of the logistics industry in each city, as shown in Table 2.

From 2005 to 2015, the average score of the logistics industry development of the cities in Table 2, the top five cities are: Shanghai, Suzhou, Hangzhou, Nanjing and Ningbo. Among them, the development level of the logistics industry in Shanghai has always been in the leading position, which may be closely related to the status of Shanghai as a mega-city; and its highly specialized logistics operation mode and more complete system of logistical talent training also provide a powerful impetus for the development of the logistics industry. At the same time, it can be seen that the last five cities include Chizhou, Xuancheng, Chuzhou, Anqing and Yancheng; most of these are located in Anhui Province, which may be related to the relatively out-dated local logistics infrastructure construction.

In addition, the average score for logistics industry development of the entire Yangtze River Delta region changed from 8.457 in 2005 to 17.927 in 2015, which shows a steady rising trend. The average annual growth rate reached 7.8%. At the same time, the average annual growth rate is different for different provinces or regions, the rate of Shanghai is 4.3%, the rate for the cities in Jiangsu province is 8.4%, the rate for the cities in Zhejiang province is 8.3%; the 8 cities in Anhui Province have the highest annual growth rate, reaching nearly 10.1%. The reason for this may be the gradual integration of Anhui into the logistical cooperative development of Yangtze River Delta urban agglomeration, in terms of logistics network planning, favorable geographical position, and the layout of urbanization.

The change of the range of values can be roughly divided into three stages: the range value increased from 48.523 in 2005 to 71.664 in 2010, indicating that the gap in the development level of the logistics industry in the urban agglomerations was widening. The range value temporarily decreased in 2010–2012, but rose from 59.849 to 71.196 in 2012–2015. The reason for this may lie in the fact that, in the early stages of the logistics industry, restricted by the resource endowment and administrative factors of the region, there is a big gap between the development levels of the inter-regional logistics industries. In the middle stage of development, growth benefits from policy support and the expansion of the regional logistics market, meaning that regional differences can be relieved. With the attenuation of policy stimulus, the development of logistics industry relies more on technological innovation and industrial upgrading, and the difference in innovation ability among regions will further aggravate the unbalanced development.

**Figure 1:**
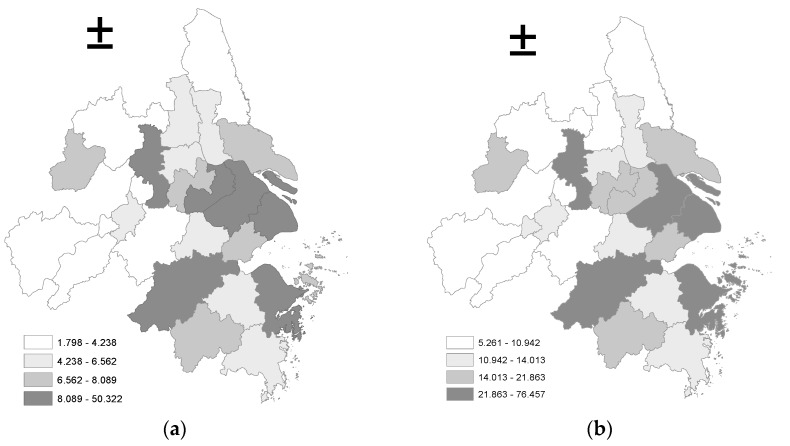
Quaternary Map of the Development of Logistics Industry in Yangtze River Delta: (**a**) 2005; (**b**) 2015.

In order to further visualize the development level of logistics industry and the spatial distribution characteristics, the ArcGIS10.2 software is used to draw the corresponding quartile-map of the starting and ending years, as shown in Figure 1.

As can be seen from Figure 1, the development level of the logistics industry in the Yangtze River Delta City Group is mainly characterized by high levels in the eastern coastal cities and low levels in inland cities in the central and western regions. Specifically, the level of logistics industry development in the first and second rank of the city is mostly located in Anhui (for example, Anqing, Tongling, Chizhou, Xuancheng, Chuzhou, etc.) and the central Jiangsu (Yangzhou), northern Jiangsu (Yancheng), and other regions. The overall level of development of the logistics industry is relatively high along the Shanghai-Nanjing railway line (Shanghai, Suzhou, Wuxi, Changzhou, Zhenjiang, Nanjing), with most of those cities hovering in the third and fourth rank of the quartile map, and the high logistics industry development level of Zhejiang is mainly in Hangzhou, Ningbo, etc.

### 3.3. Spatial Autocorrelation Test

The spatial autocorrelation of the development level and economic growth of each city was analyzed. The calculated Moran’s Index is shown in Table 3.

It can be seen from Table 2 that the Moran’s Index of the development of the logistics industry in the Yangtze River Delta from 2005 to 2015 is greater than zero, and the Moran’s Index is significant in all years other than 2005, indicating that there is a positive spatial correlation between the development level of the logistics industry in each city. The Moran’s Index of regional economic growth is positive, and all passed the 5% level of significance test, indicating that the explained variable of the model does not meet the traditional uniform distribution of the classic econometric assumptions, and there is a necessity to introduce a spatial measurement model for analysis.

### 3.4. Analysis of Spatial Spillover Effects

Multiple collinearity diagnosis is performed on the selected variables before the regression, and the correlation coefficient matrix and VIF test are calculated respectively, as shown in Table 4 and Table 5.

As can be seen from Table 4 and Table 5, the correlation coefficient between lnWL and lnL is 0.833, the correlation coefficients between the remaining variables are all less than 0.8, and the VIF test values are less than 10; therefore, there is no serious multiple collinearity problem between the model variables.

**Figure 2:**
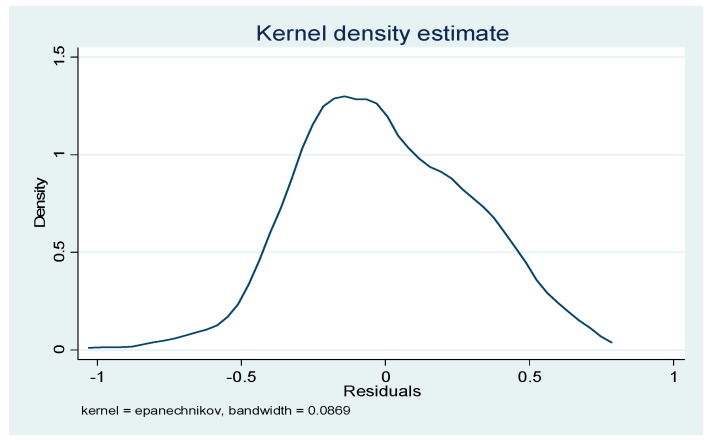
Kernel density estimation of residuals.

Regression results of non-spatial panel models and related tests are shown in Table 6 and Figure 2.

As can be seen from Table 6, the statistic of the Durbin-Watson test is 1.960, which is close to the threshold value of 2, indicating that there is no residual serial first-order autocorrelation problem. In response to the possible heteroscedasticity problems, robust standard errors are used in the model. In addition, in Figure 2, which shows Kernel density estimation of residuals, is provided by Stata12.0 software, and indicates that the residuals also approximately obey normal distributions. The joint significance of time-period and spatial fixed LR test rejects the null assumption at 1% level, which indicates that spatial fixed effect and time-period fixed effect should all be considered in the model. At the same time, the results of LM-lag and LM-error reject the null hypothesis at 1% level, Robust LM-lag passes the 1% level of significance test, but Robust LM-error fails the 10% level of significance test. So the model should add a spatial lag item, and the SAR model will be better than the SEM model.

On this basis, the applicability of SDM is tested to see if SDM can be simplified to SAR or SEM, and the ML estimation method and robust standard errors are used to estimate the coefficients of the spatial econometric model. The estimation and test results are shown in Table 7.

As can be found in Table 7, the Wald and LR tests all reject the null assumptions of $H_{0}:\theta =0$ and $H_{0}:\theta +\rho \beta =0$, at least at 5% levels, indicating that SDM cannot be simplified to SAR and SEM. At the same time, the adjusted $R^{2}$ of the SDM is 0.668, and the value of Log-likelihood is 755.181; both are obviously larger than the SAR and the SEM model’s corresponding value, which shows that the double fixed effect SDM model’s fitting effect is relatively good; therefore, this paper will carry on the analysis of the spatial and time-fixed SDM model’s regression results. Of these, the spatial lag coefficient of the dependent variable is 0.798, and it is significant at 1% level, indicating that the economic growth in the surrounding area has a significant positive spillover effect on local economic growth. At the same time, the regression coefficients of the logistic industry development level, the labor force input, and the material capital investment are significantly positive, which explains that those factors have an obvious promotion function for regional economic growth. Because the regression coefficients of the spatial lag of the explanatory variables will influence the feedback effect, the coefficients cannot be directly considered as a spatial spillover effect [23].

Therefore, the partial coefficients of the SDM model are analyzed by partial differential methods. The direct influence coefficient and the spatial spillover effect coefficient of each variable is obtained. The results are shown in Table 8.

Table 8 indicates that the direct effect coefficient of logistics industry development is 0.092 and is significant at a 1% level, which shows that the development of the logistics industry in the Yangtze River Delta has an obvious positive effect on local economic growth. At the same time, the spatial spillover effect coefficient of the logistics industry is 0.197, and it is significant at a 10% level, which indicates that the development of the local logistics industry in urban agglomeration will have a significant positive impact on the economic growth of its surrounding areas. The direct effect of material capital investment and government function on regional economic growth and spatial spillover effect are significantly positive. The direct effect of the labor force input is obviously positive, the coefficient of the spatial spillover effect is also positive, but not significant. In addition, the direct influence coefficient of external opening level and the coefficient of spatial spillover effect are negative, but none of them passed the significance test at the 10% level.

## 4. Conclusions

Based on the qualitative analysis of the spillover mechanism of logistics growth on the economic growth, this paper uses the spatial durbin model to study the direct impact of the development of the logistics industry on the regional economic growth and the spatial spillover effect of the Yangtze River Delta in 2005–2015. The results show that the direct impact coefficient of logistics industry development in the Yangtze River Delta city group is 0.092, with obvious promotion of local economic growth; spatial spillover coefficient of logistics industry development on regional economic growth is 0.197, and the local logistics industry development level increased by 1%, while surrounding regional economic growth increased by 0.197% in the urban agglomeration, representing a higher direct effect coefficient for the logistics industry on economic growth than those of labor force input, material capital investment, opening level and government function, with the spatial spillover effect having a higher coefficient than other control variables, except for material capital investment. Thus, the logistics industry is becoming the ”strong engine” of the Yangtze River Delta urban agglomeration’s economic growth.

## Figures and Tables

**Table 1:** Evaluation Index System of logistics industry development level in Yangtze River delta.

Target Level	First Grade Index	Second Grade Index (Dimension)
Development level of logistics industry	A_1_: Industry Scale	A_11_: Revenue from Postal and Telecommunications Services (million Yuan)
		A_12_: Per square km revenue from Postal and Telecommunications Services (million Yuan/km^2^)
		A_13_: Freight traffic (ton)
		A_14_: Per square km freight traffic (ton/km^2^)
	A_2_: Infrastructure	A_21_: Road density (km/km^2^)
		A_22_: Per capita car ownership (car/million persons)
		A_23_: Per square km number of mobile phone subscribers (million households/km^2^)
	A_3_: Human Resources	A_31_: Logistics employment (million persons)
		A_32_: The proportion of logistics employment account for all industrial employees (%)
	A_4_: Industrial Support	A_41_: Number of industrial enterprises above designated size (unit)
		A_42_: Number of wholesale and retail trades enterprises above designated size (unit)

Note: The statistical data are derived from the 2005–2015 China City Statistical Yearbook and the Statistical Yearbook of Cities. As China has not yet considered the logistics industry as an independent industry, the data of logistics employment are replaced by the data of transportation, storage and postal services.

**Table 2:** The score $F_{i}^{t}$ of the development level of the logistics industry.

City	2005	2008	2010	2012	2015	Average Value
Shanghai	50.322	68.610	74.809	63.523	76.457	65.793
Nanjing	13.984	16.996	20.633	23.374	28.825	20.702
Wuxi	10.322	13.703	19.565	19.772	21.863	17.063
Changzhou	7.811	12.549	15.038	16.523	18.807	14.299
Suzhou	11.461	16.466	23.914	26.169	30.077	21.681
Nantong	7.187	9.706	13.578	15.032	15.223	12.159
Yancheng	4.238	5.579	6.968	8.087	10.026	7.084
Yangzhou	5.369	6.952	8.895	10.230	10.942	8.592
Zhenjiang	6.147	8.012	9.902	11.474	11.634	9.402
Taizhou	4.610	7.125	8.813	9.805	11.845	8.359
Hangzhou	13.837	19.419	21.774	23.061	28.351	21.032
Ningbo	12.416	20.108	23.409	21.744	26.658	20.396
Jiaxing	8.089	11.895	15.616	17.204	19.383	14.447
Huzhou	5.883	7.914	9.382	10.641	11.700	9.228
Shaoxing	6.295	8.755	9.728	10.709	14.013	9.751
Jinhua	7.445	10.108	11.192	11.889	14.225	10.841
Zhoushan	7.524	10.404	13.204	16.930	22.865	13.909
Taizhou	6.562	10.280	12.160	12.207	13.564	10.940
Hefei	7.196	9.299	11.747	13.625	16.731	12.018
Wuhu	5.084	9.697	11.531	11.113	13.593	10.184
Ma’anshan	3.358	5.965	9.432	8.508	8.866	7.327
Tongling	4.183	5.372	8.106	9.988	12.088	8.336
Anqing	3.200	3.830	5.721	6.789	7.143	5.614
Chuzhou	3.284	4.387	5.370	5.798	8.660	5.520
Chizhou	1.798	2.386	3.145	3.674	5.261	3.362
Xuancheng	2.269	3.358	4.700	5.577	7.313	4.737
Average Value	8.457	11.880	14.551	15.133	17.927	-
Range	48.523	66.224	71.664	59.849	71.196	-

Note: The results are calculated using Excel 2013 and retained 3 decimals. Due to space limitations, only the scores of the representative years are listed here.

**Table 3:** The Moran’s Index of logistics industry development and regional economic growth.

Year	Logistics Industry Development (*WL*)	Regional Economic Growth (*GDP*)
2005	0.078 (1.572)	0.192 ** (2.250)
2006	0.083 * (1.677)	0.194 ** (2.255)
2007	0.113 * (1.841)	0.194 ** (2.257)
2008	0.095 * (1.735)	0.197 ** (2.251)
2009	0.108 * (1.856)	0.199 ** (2.242)
2010	0.143 ** (2.140)	0.201 ** (2.235)
2011	0.164 ** (2.127)	0.203 ** (2.222)
2012	0.185 ** (2.241)	0.204 ** (2.210)
2013	0.164 ** (2.153)	0.205 ** (2.203)
2014	0.161 ** (2.032)	0.208 ** (2.198)
2015	0.154 * (1.953)	0.207 ** (2.195)

Note: **, * represent 5%, 10% levels of significance; ** is significant at the 5% level and z statistic is in brackets.

**Table 4:** Correlation coefficient matrix of variables.

	ln*WL*	ln*Inv*	ln*L*	ln*Open*	ln*Gov*
ln*WL*	1.000				
ln*Inv*	−0.408	1.000			
ln*L*	0.833	−0.541	1.000		
ln*Open*	0.740	−0.588	0.611	1.000	
ln*Gov*	0.078	−0.239	0.275	−0.114	1.000

Note: The results of the table are calculated by Stata12.0 and retain 3 decimal places.

**Table 5:** Variance inflation factor test.

Variable	VIF	1/VIF
ln*WL*	5.27	0.189
ln*Inv*	4.41	0.227
ln*L*	3.49	0.287
ln*Open*	2.10	0.476
ln*Gov*	1.38	0.726

Note: The results of the table are calculated by Stata12.0.

**Table 6:** Results of non-spatial panel model regression and related tests.

Variable	Estimated Value	T Value/Statistic	*p* Value
ln*WL*	0.114 **	2.685	0.013
ln*L*	0.011	0.692	0.495
ln*Inv*	0.122 ***	5.803	0.000
ln*Open*	0.002	0.108	0.915
ln*Gov*	0.053 *	1.713	0.099
Adjusted R^2^	0.430		
Durbin-Watson test		1.960	
Log-likelihood	598.378		
LM-lag		220.865 ***	0.000
Robust LM-lag		73.228 ***	0.000
LM-error		147.628 ***	0.000
Robust LM-error		0.001	0.994
LR-test joint significance spatial fixed effects, (degree of freedom)		1281.909 ***(26)	0.000
LR-test joint significance time-period fixed effects, (degree of freedom)		582.917 ***(10)	0.000

Note: The results of the table are calculated by Stata12.0, and retain 3 decimal places. ***, **, * represent 1%, 5%, 10% levels of significance.

**Table 7:** Estimated results of spatial econometric models.

Variable	Spatial and Time Fixed SDM	Spatial and Time Fixed SAR	Spatial and Time Fixed SEM
ln*WL*	0.071 *** (2.774)	0.067 *** (2.675)	0.059 ** (2.498)
ln*L*	0.014 ** (2.535)	0.013 ** (2.014)	0.009 (1.192)
ln*Inv*	0.047 *** (3.232)	0.050 *** (3.159)	0.043 ** (2.593)
ln*Open*	0.006 (0.850)	0.008 (0.788)	0.012 (1.430)
ln*Gov*	0.019 (1.458)	0.016 (1.262)	0.019 (1.304)
W * ln*WL*	−0.014 (−0.639)		
W * ln*L*	0.004 (0.491)		
W * ln*Inv*	0.012 (0.703)		
W * ln*Open*	−0.024 (−1.397)		
W * ln*Gov*	0.022 (1.014)		
ρ/λ	0.798 *** (16.163)	0.797 *** (12.391)	0.886 *** (21.480)
$\mathrm{Adj}\;R^{2}$	0.668	0.629	0.402
Log-likelihood	755.181	748.307	736.756
Wald and LR test	Estimated Value	*p* value	
Wald_spatial_lag	12.230 **	0.014	
LR_ spatial_lag	13.756 **	0.017	
Wald_spatial_error	40.549 ***	0.000	
LR_spatial_error	37.064 ***	0.000	

Note: the table is calculated by Stata12.0, ***, **, * represent 1%, 5%, 10% levels of significance, respectively, with Z values shown within the brackets. The results retain three decimal places.

**Table 8:** The direct effect of the development of logistics industry on regional economic growth, spatial spillover effect and total effect.

Variables	Direct Effect	Space Spillover Effect	Total Effect
ln*WL*	0.092 *** (3.167)	0.197 * (1.867)	0.289 ** (2.429)
ln*L*	0.022 *** (2.801)	0.077 (1.234)	0.099 (1.455)
ln*Inv*	0.071 *** (4.172)	0.240 ** (2.131)	0.311 ** (2.540)
ln*Open*	−0.001 (−0.099)	−0.074 (−0.806)	−0.075 (−0.737)
ln*Gov*	0.037 ** (2.217)	0.178 * (1.649)	0.215 * (1.820)

Note: this table is calculated using Stata12.0, ***, **, * represent 1%, 5%, 10% significance levels, respectively, with Z values shown in brackets, and the results retain three decimal places.
